# Reply: Remarks on sentinel node biopsy in head and neck cancer

**DOI:** 10.1038/sj.bjc.6602294

**Published:** 2005-01-14

**Authors:** S Hoft, S Maune

**Affiliations:** 1 Department of Otorhinolaryngology, Head and Neck Surgery, University Hospital Schleswig-Holstein, Campus Kiel, Arnold-Heller-Str 14, 24105 Kiel, Germany

Sir,

Thank you for giving us the opportunity to respond to the commentary letter by Dr Kovacs.

In the third paragraph, Dr Kovacs is referring to staging methods. He compares four publications (Kovacs *et al*, 2001, 2004a, 2004c; Ross *et al*, 2004) to our present publication in the British Journal of Cancer (Höft *et al*, 2004). One of the four studies was published after ours (Ross *et al*, 2004). Two are not published yet, but are in press (Kovacs *et al*, 2004a, 2004c). Dr Kovacs' conclusion that ‘sentinel lymph node biopsy reflected the accuracy of the clinical and radiological staging methods, and the ideal diagnostic prerequisite for SNB is not yet found’ is quite similar to ours, namely the ‘better the staging methods are in detecting small metastases the less occult metastases will be overlooked and the more valuable will be the impact of an additional sentinel lymph node procedure’. However, the rate of positive sentinel lymph nodes is not only influenced by the staging procedure but also by the pathohistologic work-up. The more the intensive sentinel lymph nodes are examined, the more the occult metastases will be detected, raising the percentage of patients with occult disease (Höft *et al*, 2002; Höft *et al*, 2004). In our study, we applied ultrasound examinations for staging of the neck as a radiological method and also performed fine-needle aspiration cytologies. The accuracy of US-guided aspiration cytology has been shown to be significantly better than that of CT or MRI (van den Brekel *et al*, 1991).

The fourth paragraph concerns the obstacle of performing a sentinel node biopsy on large tumours. In our group of patients, this is due to the fact that it was difficult to perform an endoscopic peritumoral injection on large tumours located in the pharynx and the larynx (Höft *et al*, 2004). Dr Kovacs adds that large tumours pose problems due to destruction of the lymphatic drainage, but he fails to provide data or literature on which his opinion is based. As we did not perform histologic studies of the lymphatics, we cannot add new information whether large tumours are more aggressive in destroying lymphatic vessels on their rim than are small tumours. However, the technique of the peritumoral injection is to inject the tracer adjacent to, and not into, the tumour. Thus, it does not really matter if a tumour destroys lymphatic vessels within its borders, as there will be intact lymphatics surrounding the tumour to take up the tracer even in large carcinomas.

In his last sentence of the fourth paragraph, Dr Kovacs suggests that an intra-arterial induction chemotherapy might be a modality in reducing tumour size prior to sentinel node biopsy. It is an interesting suggestion we would have liked to discuss in our article. However, Dr Kovacs' results were still in press when his letter of comment was written. Thus, the information he refers to was not accessible to us. Yet, although Dr Kovacs states that intra-arterial chemotherapy did not seem to alter lymphatic drainage (Kovacs *et al*, 2004b), there is ample evidence that preoperative chemotherapy affects lymph nodes. Cohen *et al* (2000) describe lymph nodes showing areas of fibrosis, fat necrosis, histiocytic accumulation and granulation formation after neoadjuvant chemotherapy. Furthermore, metastatic foci can be completely obliterated by chemotherapy. Accordingly, Nason *et al* (2000) concluded in their study that preoperative chemotherapy is associated with an unacceptable high false negative rate for sentinel lymph node detection. If these changes are evident in chemotherapy, we would expect to find them in patients with intra-arterial chemotherapy, too. We are looking forward to the publication of Dr Kovacs' results and histologic findings of the lymphatics.

In the fifth paragraph, Dr Kovacs refers to the omission of an elective neck dissection potentially achieving a benefit for the patient in respect to risk, morbidity and quality of life. He complains that Ross *et al* (2002) were not the first to publish a true sentinel node biopsy, but Dr Kovacc himself (Kovacs *et al*, 2001). Furthermore, he is citing three articles by himself that have not been published yet (Kovacs *et al*, 2004a, 2004b, 2004c), mentioning 80 patients who underwent a sentinel node biopsy. At the point of time we wrote and published our article, only the first publication was available in the German literature (Kovacs *et al*, 2001). Dr Kovacs' method included a therapy of the primary tumour prior to sentinel node biopsy by performing an intra-arterial chemotherapy in 12 of 15 patients. In his article, the author did not discuss the problem of chemotherapy causing changes in the lymphatic drainage of the tumour. Yet, as discussed above, there was literature pointing out the disadvantages of prior chemotherapy. Therefore, we are of the opinion that unless proven otherwise, the methodical approach of treating the primary tumour prior to sentinel node biopsy bears the risk of false negative results.

If an elective therapy of the neck such as neck dissection or radiotherapy is omitted in favour of a true sentinel node biopsy, it is with the intent of causing less morbidity and achieving more quality of life for the patient. However, since a true biopsy of the sentinel node is not yet established for head and neck cancer, we formulated as a prerequisite that patients with a mere biopsy of the sentinel nodes should have equal regional control rates as patients after elective therapy of the neck. This mandates that no additional elective therapy is performed before or after sentinel node biopsy. Otherwise the rate of recurrences after mere sentinel node biopsy does not only present the quality of staging by sentinel node biopsy but also the quality of elective therapy of the neck. In his group of patients, Dr Kovacs performed an elective radiochemotherapy of the neck after sentinel node biopsy applying 51.3 Gy and docetaxel (25 mg m^−2^ once a week) over a period of 5 weeks (Kovacs *et al*, 2001). It is our opinion that the method designed by Dr Kovacs will result in biased data. His method does not represent a true sentinel node biopsy and is likely to discredit the sentinel node concept. Therefore, we cited Ross *et al* (2002) as they indeed were the first to publish a study on a true sentinel node biopsy without prior therapy of the primary tumour and postoperative radiotherapy of the lymphatics in the international literature.

We are still cautious about a true sentinel node biopsy in head and neck cancer and are looking forward to long-term results in comparison to elective selective neck dissection.
